# Improving Fertilizer Use Efficiency—Methods and Strategies for the Future

**DOI:** 10.3390/plants12203658

**Published:** 2023-10-23

**Authors:** Przemysław Barłóg

**Affiliations:** Department of Agricultural Chemistry and Environmental Biogeochemistry, Poznan University of Life Sciences, Wojska Polskiego 71F, 60-625 Poznan, Poland; przemyslaw.barlog@up.poznan.pl; Tel.: +48-618-48-77-93

**Keywords:** ammonia volatilization, controlled-release fertilizers, crop genotypes, elemental sulfur, magnesium, nitrogen use efficiency indices, phosphorus, potassium, root architecture, sustainability, Soil Fertility Clock

## Abstract

This editorial introduces our Special Issue entitled “Improving Fertilizer Use Efficiency—Methods and Strategies for the Future”. The fertilizer use efficiency (FUE) is a measure of the potential of an applied fertilizer to increase the productivity and utilization of the nutrients present in the soil/plant system. FUE indices are mainly used to assess the effectiveness of nitrogen (N), phosphorus (P), and potassium (K) fertilization. This is due to the low efficiency of use of NPK fertilizers, their environmental side effects and also, in relation to P, limited natural resources. The FUE is the result of a series of interactions between the plant genotype and the environment, including both abiotic and biotic factors. A full recognition of these factors is the basis for proper fertilization in farming practice, aimed at maximizing the FUE. This Special Issue focuses on some key topics in crop fertilization. Due to specific goals, they can be grouped as follows: removing factors that limit the nutrient uptake of plants; improving and/or maintaining an adequate soil fertility; the precise determination of fertilizer doses and application dates; foliar application; the use of innovative fertilizers; and the adoption of efficient genotypes. The most important nutrient in crop production is N. Hence, most scientific research focuses on improving the nitrogen use efficiency (NUE). Obtaining high NUE values is possible, but only if the plants are well supplied with nitrogen-supporting nutrients. In this Special Issue, particular attention is paid to improving the plant supply with P and K.

## 1. Introduction—Why Fertilizer Use Efficiency Should Be Improved

According to forecasts, 9.7 billion people will be living on Earth by 2050, and about 10.4 billion by 2100 [1]. Right now, the world has the resources to feed a population of 8 billion. It is, therefore, necessary to seek optimal solutions in both the political and economic areas in order to solve the problem of the ever-growing demand for food. The expansion of agricultural areas at the expense of forests or shrubs, or even barren lands, either requires too much investment or is too risky in terms of the environment and the functioning of the global ecosystem [2,3,4]. Hence, the only rational direction for agricultural development is to maximize yields from the area already covered by agricultural activity [5,6]. There are some factors that are considered crucial in activities towards yield increase: breeding progress, the effective use of mineral fertilizers and crop protection measures, and farmers and their advisers having sufficient knowledge and skills [7]. The consumption of nitrogen fertilizers plays a special role in achieving this objective [8]. Mogollon et al. [9] presented several simulations showing that the global N input in agriculture in 2050 may fluctuate widely, ranging from 87 to 260 Tg N yr^−1^. One of the main factors differentiating the above range is the nitrogen use efficiency (NUE). Currently, it is assumed that recovery of N from applied fertilizers is at the low level of just 30–50% [10]. As a result, N that is not taken up by plants is dispersed into the environment, reducing the economic profitability of agricultural production and, at the same time, causing a number of adverse changes in the functioning of the biosphere. The most important of these concern such phenomena as greenhouse gas and ammonia emissions, the destruction of the ozone layer, the eutrophication of the environment, or the impoverishment of ecosystems in plant and animal species [11]. An increase in the NUE value can be achieved through the improvement of N fertilization technology, including the use of innovative solutions in the production technology and chemical composition of fertilizers [12]. An important factor shaping the NUE is the presence of appropriate amounts and forms of minerals in the soil, which support the uptake and processing of N into plant crops [7]. This objective can be achieved using P, K, Mg, S, and other fertilizers, separately, or together with N in compound fertilizers. Hence, the term “NUE” can be extended to include the concept of fertilizer use efficiency (FUE). Such a definition allows for a broader approach to the issues related to the effectiveness of the application of all fertilizers and is not limited to only one nutrient. The aim of this Special Issue is to present the latest knowledge and research results regarding the improvement of the FUE/NUE in the cultivation of various plant species.

## 2. Special Issue Overview—General Topics

### 2.1. Factors Effecting Fertilizer Use Efficiency (FUE)

The first chapter of the Special Issue comprises two papers that focus on factors limiting the uptake and use of nutrients from fertilizers by crops, as well as on the present strategies and prospects for improving fertilizer use efficiency [12]. The term fertilizer use efficiency (FUE) is not new. It has been widely used for decades, but has become widespread thanks to the use of the FUE indices to assess the global productivity of NPK fertilizers. The number of indices used to characterize the FUE is vast, and their choice depends on the purpose of the analysis and/or comparisons [10,13]. The first article in the presented series of publications shows the concepts and principles of calculating a relatively new index, which is the nitrogen gap (NG). The NG calculation is important for the identification of hotspots in N management for a given crop, including the inadequate supply of nutrients other than N and a set of activities needed to improve the level of soil fertility for a given crop [14,15]. The impact of soil factors on the FUE should be considered as several groups of phenomena and processes [12]. The first group refers to factors affecting the nutrient uptake. However, there is a major challenge for a farmer to synchronize the crop plant requirement for nutrients with their supply from both soil and applied fertilizers. Achieving this goal requires extensive knowledge of plant growth dynamics and the critical phases of crop formation. After recognizing the nutritional requirements of plants, another area of activities aimed at improving the FUE is to create optimal conditions for plant root growth and eliminate all factors limiting the inflow of nutrients to the root surfaces. The most important soil factors shaping the uptake of nutrients and the FUE are the soil texture, water content, soil compaction, temperature, soil reaction, salinity, soil organic matter, and nutrient shortage [12]. Among them, the soil compaction and pH are relatively easy to correct in agricultural practices. The presented literature shows that FUE values can be shaped by building appropriate root architecture (RSA) in crops. This is possible by applying proper fertilization with N and K [16]. Another way to improve the FUE is the use of new and innovative fertilizers [12].

The second overview article presents the concept of effective fertilization, defined as the Soil Fertility Clock (SFC) [7]. At the core of this concept, there are three basic facts: (i) a crop plant in a well-defined geographic area, provided with stable environmental and nutritional conditions, can reach maximum yield (Y_max_); (ii) the key production factor is N, present in the soil or/and supplied to the plant as fertilizer (organic and mineral, N_f_); (iii) all other nutrients, called nitrogen-supporting nutrients (N-SNs), affect the Y_max_, in relation to their relative deficiency in available form in the plant rooting zone. The classic concepts of N-SNs do not take into account that crop plants differ in their sensitivity to the supply of N-SNs in two crucial aspects: during the growing season and in the course of crop rotation. The Soil Fertility Clock (SFC) is an approach based on three assumptions: (i) the critical soil fertility is the value or range of soil nutrient content that is sufficient to provide an appropriate amount to the plant most sensitive to its supply in a given crop rotation; (ii) the non-sensitive plants in the given crop rotation create the necessary timeframe for the recovery of its original critical content; and (iii) the content of a specific nutrient cannot be a limiting factor in N uptake and utilization for any crop grown. The SFC concept is supported primarily by the yield-promoting role of P and K [7]. A deficiency of both nutrients in the soil during the critical stages of yield formation results in a decreased N_f_ efficiency, and consequently, a lower yield. Thus, the main goal of P and K application to the soil is to restore their content in the topsoil to the level required by the most sensitive crop in any given crop rotation.

### 2.2. Improving FUE by Optimizing N Uptake and Rate

One of the most important activities aimed at improving the FUE is the correct selection of the fertilizer dose for specific soil and climatic conditions, the applied agrotechnics, and the plants requirements in crop rotation. This can be achieved using analytical tools such as soil testing, plant tissue analysis, nutrient uptake dynamics, fertilizer rate response modeling, or digital and information technologies [7]. The standard methodology for determining the need for N fertilizers is based on data regarding the mineral nitrogen (N_min_) content in the soil [17]. Therefore, it is extremely important to identify and classify the factors affecting the mineralization processes of organic nitrogen and the N_min_ content in the soil. The knowledge gained in this area can be translated into conscious control of N_min_, thus shaping the yield level. The first article included in the subsection discusses the influence of various tillage practices on the content of different forms of N in fluvo-aquic soil from Huang-Huai-Hai Plain in China [18]. The experiment evaluated the effect of five treatments where rotary tillage (RT), deep tillage (DT), and shallow rotary tillage (SRT) were used. The test plant was wheat. The results showed that the rotation tillage with deep tillage increased the total N and the content of the mineral nitrogen forms compared with RT-RT-RT. They especially improved the NO_3_–N and NH4–N content in 0–40 cm, with the highest value under DT-SRT-RT. However, the effect of deep tillage on dissolved organic N in deeper layers significantly declined with time. The highest wheat yield was under DT-SRT-RT in 2018 and 2019, with 6346 and 6557 kg ha^−1^, respectively. The N partial productivity demonstrated a similar trend with the wheat yield, with higher values of 28.98 and 29.94 kg^−1^, respectively. The authors also obtained the lowest apparent nitrogen loss values in the DT-SRT-RT treatment. It was suggested as the efficiency tillage practice to improve the NUE and the crop yield [18].

In field conditions, plants compete with each other for water and nutrients. Therefore, it is important to recognize the appropriate sowing density (SR) to minimize these effects and, at the same time, consciously combine yield components to obtain the maximum N productivity. The problem of the NUE’s dependence on the sowing density in winter wheat cultivation in Jiangsu province (China) was analyzed by Mahmood et al. [19]. The authors put forward the hypothesis that there is an optimum seed rate to compensate the negative effects of decreasing N for balanced high yields and an improved NUE in wheat. The results revealed that the net photosynthetic rate, the stomatal conductance, the chlorophyll content, and the activities of metabolic enzymes significantly increased with increasing N levels and a decreasing seeding rate. The plant tillers, grain yield, dry matter before anthesis and N translocation, N agronomic efficiency (NAE), N recovery efficiency (NRE), and N uptake efficiency (NUPE) were highest in a combined treatment of N235 and SR180. However, N levels beyond 235 kg ha^−1^ significantly decreased the NAE, NRE, and NUPE. The authors concluded that 1 kg N ha^−1^ might be replaced by an increase of approximately 0.6 kg ha^−1^ SR. In addition, by using a combination of N and SR (N235 + SR180), it is possible to obtain the maximum yield of winter wheat and improve the NUE parameters [19].

The objective of another paper was to determine the best pruning level and N dose based on the agro-physiological characteristics of kaffir lime under mild shading [20]. The research was based on the need to fill the information gap regarding growth and yield under mild-shading conditions and specific N recommendations for leaf-orientated production of kaffir lime. The experiment was carried out at the Pasir Kuda experimental field of IPB University, Bogor, Indonesia. The plant materials were nine-month-old seedlings obtained using a grafting technique that combined kaffir lime (*Citrus hystrix* DC) scions onto rangpur lime (*C. limonia* Osbeck) rootstock. Four levels of N dosage were tested. The optimum N rate was determined based on a regression curve. A N-sufficient condition was achieved as the effect of 20 and 40 g N plant^−1^ application, producing a great growth and yield performance due to a high carbon assimilation rate. However, that does not automatically mean that a dose of 40 g N plant^−1^ is the best fertilizer recommendation, as 20 g N plant^−1^ is more efficient, with a relatively similar effect for increasing kaffir lime leaf production. With respect to pruning, a higher yield was obtained via leaving 30 cm of main stem above the ground, rather than shorter plants with a 10 cm main stem [20].

### 2.3. Balanced Fertilization as Key to Efficient N Use

Efficient N uptake, transport, and conversion into a crop depends on a good supply of plants with the remaining macro- and micronutrients [7]. The first publication dedicated to balanced fertilization described their results regarding fertilization in a rice–rice cropping system [21]. As rice is a nutrient-exhausting crop, its properly balanced fertilization is important to maintain a high productivity. The two-year experiment in a sub-tropical climate under the red and lateritic belt of the western part of West Bengal, India, was set up in a randomized complete block design with twelve treatments and three replications, with different rates of N:P:K:Zn:S application in both of the growing seasons, namely, Kharif and Boro. The results clearly indicated that imbalanced or insufficient nutrient application affects crop nutrient removal, thus affecting the growth and development of the plant. In addition, inappropriate nutrient supply over a long period reduces soil fertility, especially when a nutrient-exhausting cropping system, such as a rice–rice cropping system, is chosen. In this study, the recommended dose of nutrients was 80:40:40:25:20 and 120:60:60:25:20 kg ha^−1^ of N:P_2_O_5_:K_2_O:Zn:S in the Kharif and Boro season, respectively. To summarize, balanced nutrient management in cropping systems is a cost-effective and environmentally friendly approach to targeting agricultural sustainability [21].

In another paper, the authors focused on interactions between differentiated fertilization management and environmental factors and their influence on potato yields and selected soil parameters [22]. The fertilization treatments represent different management practices and include: (1) an unfertilized control, (2) the application of cow manure (FYM), (3, 4) a combination of manure and two different mineral nitrogen rates (FYM + N1, FYM + N2), and (5, 6, and 7) a combination of FYM and mineral NPK fertilizers (FYM + N1PK, FYM + N2PK, FYM + N3PK), which represents the combination of manure and all three major mineral fertilizers (against FYM + N treatments). The experiment was carried out on three sites (different soils) and during four growing seasons. Both the growing season and fertilization significantly affected potato yields at all locations. The authors proved that FYM application was always associated with higher yields. However, FYM application did not provide enough nutrients (N) to fulfil the yield potential of potatoes. Therefore, the addition of mineral N significantly increased potato yields, especially at less-fertile sites. The FYM + NPK combinations significantly improved yields compared to the FYM + N treatments. Thus, the obtained results clearly confirm the important role of P and K fertilization in increasing N productivity via both natural and mineral fertilizers.

The role of balanced fertilization in yield formation was also analyzed via two long-term experiments. The first was set up in 1954 in Prague and analyzed the effect of weather and seven fertilization treatments (mineral and manure treatments) on winter wheat grain yield and stability [23]. Winter wheat is one of the most important crops in the world. Hence, analysis of the response of wheat varieties to perennial fertilization is particularly important for food security. The authors analyzed 23 growing seasons. They showed that the grain yield was positively associated with the April precipitation, the mean daily temperature in October, and the daily maximum temperature in February. The yields were most stable between years when two fertilizer treatments were used that supplied a mean of 47 kg N ha^−1^ yr^−1^, 54 kg P ha^−1^ yr^−1^, and 108 kg K ha^−1^ yr^−1^. The rate of N at which the grain yield was optimized was determined according to the linear-plateau (LP) and quadratic response models as 44 kg N ha^−1^ yr^−1^ for the long-strawed varieties and 87 kg N ha^−1^ yr^−1^ for the short-strawed varieties.

Another article included in this subsection presents the impact of well-balanced fertilization on the effective N fertilization of corn [14]. The objective of the study was the influence of the band application of a di-ammonium phosphate and ammonium sulfate mixture (NPS) on the possibility of lowering the total N dose. In order to assess the impact of fertilizing agents, seven nutrient efficiency indices and eight dry matter and N management indices were used. The total N uptake and NUE indices increased after band application. In addition, a trend of improved N remobilization efficiency and the N contribution of remobilized N to grain as a result of the band application of NP(S) was observed. The most effective use of N by corn was ensured via the use of an NPS mixture during the sowing of corn seeds (band application). From the point of view of the NUE indices, the optimal dose of N was 60 kg ha^−1^. With broadcast fertilization and/or a further increase in the N dose, without the simultaneous use of P and S, the values of the NUE indices deteriorated, especially in the year with the highest content of N_min_ in the soil. Thus, a positive effect of the interaction of N and P(S) was confirmed in the conditions of soil rich in plant-available P.

Another publication concentrated on the improvement of N use by potato plants through the additional application of elemental sulfur, S^0^ [24]. Potatoes require a good supply of S^0^ for effective growth. Earlier studies showed that, in conditions of good S supply, a simultaneous increase in the NUE was noticed [25]. In this study, two main goals were set: (i) quantify the seasonal growth trends in the biomass of potato organs competing with tubers and (ii) evaluate the impact of S^0^ on the in-season relationships within the biomass of potato organs. The research factors were two doses of N (60 and 120 kg ha^−1^), elemental sulfur fertilization (control and 50 kg ha^−1^), and different plant sampling dates (10-day intervals). It was found that the potato growth pattern coded at the onset of tuberization was a decisive factor for the dry matter partitioning between the potato organs during the subsequent tuber growth phase. The tuber yield-forming effect of added sulfur results from a balanced growth in stems during the ascending and the descending phase. At harvest, the average biomass of potato tubers on the main plot fertilized only with N was lower by 21% than that on the one receiving sulfur at the rate of 50 kg ha^−1^.

In a methodological publication by Hu et al. [26], a hypothesis was formulated that the optimal fertilizer doses can be determined via yield–fertilizer rate response modeling. For this purpose, the authors analyzed dozens of experiments with peanut plants located on the North China Plain. Two fertilization treatments, namely, that used by farmers (FP) and optimized fertilization (OPT), allowed for the regional mean optimal rate (ROMR) method to be applied. The authors determined the optimum fertilizer rate using the 2^o^ regression curve. In order to assess the fertilization effectiveness, the authors used a number of indices: the RIEN (N reciprocal internal efficiency), PFPN (N fertilizer partial factor productivity), NUpE (N uptake efficiency), and NUtE (N utilization efficiency). The results of the experiments supported the hypothesis that the FP treatment with the OPT treatment, based on the RMOR method, promoted N use efficiency (PFPN and NUPE) and decreased the nutrient inputs from chemical fertilizer, especially N and P fertilizers, without the loss of peanut yield and NPK uptake. The research clearly shows that the RMOR method can be adopted in many countries and regions with widespread smallholder farms.

### 2.4. FUE and Foliar Fertilization

One way to provide plants with nutrients during the vegetative phase is foliar fertilization. This treatment allows for interventional (when deficiency symptoms appear) or preventive fertilization, taking into account the growth phases in which plants show the greatest sensitivity to nutrient deficiency. The method bypasses the stage of the transformation of nutrients in the soil, and thus reduces the potential regression of components and/or dispersion into the environment. In addition, through the use of small doses, a high fertilizer productivity is achieved [27]. There are insufficient data in the literature on foliar fertilization with phosphorus and, in particular, on plants of the *Fabaceae* family. The results published in this Special Issue broaden our knowledge on foliar P application and its influence on selected growth parameters, the production, and the quality of peas [28]. The effect of foliar P application on the photosynthetic parameters, seed yields, and quality of four pea genotypes (two normal-phytate cultivars and two low-phytate) was investigated in a pot experiment in controlled conditions. The effect of the pea lines on the foliar P fertilization was different. In the case of the normal-phytate cultivars, the seed production was enhanced via gradual doses of the P-fertilizer, except for the highest dose of phosphorus (P3). Low-phytate cultivars showed a positive reaction to the P3 dose. The authors concluded that foliar P application could be an effective way to enhance the pea growth in the P-deficient condition, with a direct effect on the seed yield and quality.

The research objective of another publication was to verify the effect of the foliar application of waste elemental sulfur (S^0^) from biogas production in combination with conventional liquid UAN fertilizers applied in different ratios [29]. The reaction in maize was studied via a pot experiment. The following fertilization treatments were studied: control, UAN, UANS1 (N:S ratio, 2:1), UANS2 (1:1), and UANS3 (1:2). It showed that the application of UAN increased the N content in the plant and significantly affected the chlorophyll content (the N-tester value). The application of UANS had a lower impact on the N content and uptake than the application of UAN; however, it had a significant effect on the quantum yield of PSII. The authors conclude that the foliar application of UAN fertilizer in combination with S^0^ in a 1:1 ratio seems to be a sensible way to optimize the nutritional status of maize, both in terms of the economics of biogas purification, where the waste sulfur is reused as a fertilizer, and for environmental reasons.

Apart from P and S, the most important component for N uptake and metabolism in plants is Mg. In agricultural practice, farmers use two basic Mg fertilization systems: (i) the in-soil application of Mg fertilizer using lime for acidic soils and magnesium sulfate for soils with an optimum pH; and (ii) foliar fertilization. In studies carried out by Potarzycki et al. [30], a hypothesis was formulated that winter wheat fertilized with Mg increases nitrogen fertilizer (N_f_) efficiency, regardless of the method of application. In order to achieve this, the authors set a two-factorial experiment with three doses of Kieserite (0, 25, and 50 kg ha^−1^ of Mg) and two stages of foliar fertilization at the rate 2.4 kg Mg ha^−1^ (control; I; II; I + II). A full dose of nitrogen was 190 kg ha^−1^. Twelve different parameters and indices (the total N accumulation, harvest index, partial factor productivity, nitrogen physiological efficiency, and others) were used to assess the impact of factors on the nitrogen efficiency (NUE) in wheat cultivation. The same set of indicators was used to assess the effectiveness of Mg fertilization. According to the study, the wheat yield increased as a result of the use of Mg. The method of application was of secondary importance. The yield gain, as a result of foliar fertilization with Mg fertilization, ranged from 0.6 to 0.9 t ha^−1^, while, in the soil, its application resulted in a yield gain in the range of 0.4–0.7 t ha^−1^. The main action of Mg, regardless of its application method, was the improvement of the index values characteristic for the NUE. The yield-forming effect of the applied Mg on the winter wheat was revealed via the increased N transfer to the grain.

In another publication included in this Special Issue, the authors investigated the effect of three foliar fertilizers (F, B, and C) and the mixture of the three (F + B + C) on the flower quality and the amount of new daughter corms produced by the five Gladiolus varieties in the climate conditions of the Carpathian Basin [31]. The *Gladiolus* genus is a perennial, monocotyledonous, geophyte, semi-rustic ornamental plant and includes about 260 species [32]. These plants are valued for the variety of shapes and colors of their flowers. However, they require appropriate growing conditions and the correct selection of varieties, in particular for degraded and saline soils. In the study, the authors used multicomponent foliar fertilizers that differed not only in the set of elements and their content but also in the presence of phytohormones. It should also be mentioned that N was included in each fertilizer. During the season, a total of four sprayings were carried out during plant development phases. The results of this experiment show that proper foliar fertilization can support and influence the growth, vase durability, and daughter corm production of some Gladiolus varieties. The highest yield of daughter corm production was observed with the mixture of the three foliar fertilizations (F + B + C). The result confirms that N productivity is stimulated not only via the dose of N but also via the appropriate balance of all nutrients [31].

### 2.5. FUE and Innovations on the Fertilizer Market

For many years, mineral fertilizers have been used to (i) ensure a good supply of nitrogen to plants, especially in critical phases; (ii) reduce the number of applications; (iii) reduce the nitrate content in plants; and (iv) limit nitrogen loss and reduce its negative impact on the environment [12]. In general, these fertilizers can be divided into two groups: slow-release fertilizers (SRFs) and controlled-release fertilizers (CRFs). With regards to N fertilizers from the CRF group, the effect of delaying N release is achieved through covering the granules with a different type of protective layer. Škarpa et al. [33] assessed the possibility of improving the efficiency of N_f_ and reducing its negative impact on the environment (N leaching) through the use of two CRF fertilizers: calcium ammonium nitrate (CAN) fertilizer coated with modified conventional polyurethane and CAN coated with vegetable oils. The influence of the CRF fertilizer was compared to that of the classic CAN. Three types of treatment were tested for both coated fertilizers: divided application (CAN, coated CAN), a single application of coated CAN, and a single application of CAN with coated CAN (1:2). The test plant was winter oilseed rape. The obtained results confirm that the application of coated CAN fertilizers increases the yield to a large extent, improves the efficiency of N fertilization, and reduces N losses, compared to the use of conventional CAN. In this study, a suitable method appears to be the application of a mixture of conventional CAN and coated CAN in a ratio of 1:2 during spring fertilization, ensuring a sufficient amount of rapidly releasing N during the regeneration of rapeseed and its slower release during further developmental stages. In terms of fertilizer production, oil-based polymer coatings on CAN fertilizer can be considered as an adequate replacement for partially modified conventional polyurethane [33].

The second publication on CRF fertilizers in this collection studied the possibility of enhancing the NUE in coffee cultivation (*Coffea arabica* L.). Freitas et al. [34] formulated a hypothesis that enhanced-efficiency N fertilizers and other fertilizers, such as ammonium nitrate and sulfate and prilled urea diluted in water, are options more suitable than conventional urea for reducing NH_3_-N losses in coffee production systems. In order to validate the hypothesis, field experiments were carried out, in which the authors tested the following fertilization treatments: prilled urea, prilled urea dissolved in water, ammonium sulfate (AS), ammonium nitrate (AN), urea + Cu + B, urea + adhesive + CaCO_3_, and urea + NBPT (all with three split applications), as well as blended N fertilizer, urea + elastic resin, urea-formaldehyde, and urea + polyurethane (all applied only once). The experiment with fertilizer treatments was conducted in coffee plantations in field conditions for two crop seasons in the Minas Gerais region, in Brazil. The treatments used in this study were applied at the 300 kg N ha^−1^ dose per year. The authors proved a significant influence of various fertilization combinations on urea losses. Except for urea + adhesive + CaCO_3_ (27.9% of NH_3_-N losses), all N-fertilizer technologies reduced NH_3_-N losses compared to prilled urea. The lowest losses were observed for AS (0.6%) and AN (0.5%). The authors point out, however, that when choosing the right fertilization strategy (choice of treatment), the costs of the fertilizer application must be considered.

The problem of reducing losses of NH_3_-N from fertilizers was also studied by Cassim et al. [35]. The authors assessed different nitrogen (N) fertilizer technologies in corn production systems through the characterization of N sources, NH_3_-N volatilization losses, and their effects on the nutrient concentration and yield of corn grown in clayey and sandy soils in south Brazil. The following treatments were tested: control, three conventional N sources (urea, ammonium sulfate, and ammonium nitrate + calcium sulfate), and three efficiency-enhanced fertilizers (urea treated with NBPT + Duromide, urea formaldehyde, and polymer-coated urea + urea treated with NBPT and nitrification inhibitor, NI). The article features the physical properties of fertilizers obtained using scanning electron microscopy and X-ray diffraction. In general, the effect of N fertilizer technologies on N losses via the volatilization of NH_3_-N was ordered as follows: urea > URP + Ur-NBPT + IN > Ur-NBPT + Duromide > Ur-formaldehyde > ammonium nitrate + calcium sulfate > ammonium sulfate. The studies confirmed that ammonium sulfate and ammonium nitrate have the least impact on NH_3_-N losses (84 and 80% in relation to urea). Additionally, both fertilizers increased the corn grain yield. The yield increase in the clayey soil did not occur solely due to the reduction in losses via NH_3_-N volatilization. Other factors, such as S and B supplementation and N release at a controlled rate to synchronize with the crop demand, also influenced the increase in corn yield. The authors also presented interesting data regarding the effect of fertilizer treatments on the macro-i micronutrient content and the chlorophyll concentration (SPAD) at the R1 phenological stage (silking). The results suggest that the use of nitrification inhibitors in soil, which leads to an increased concentration of NH_4_^+^, primarily reduces the uptake of Ca^2+^, and then Mg^2+^, to a lesser extent.

Biochars constitute a relatively new fertilizer on the market. In general, biochars are solid materials, rich in carbon, obtained from the thermochemical decomposition of organic biomasses. They may be treated as mineral fertilizers or as a component for the production of CRF fertilizers [12]. The in-soil application of biochars has a positive impact on carbon sequestration in soil and on reducing greenhouse gas emissions [36]. In addition, biochar application improves soil fertility and crop productivity. However, the literature does not provide sufficient data on the effect of biochars on the physiology of tomato yields. This gap is filled by the publication by Liu et al. [37]. The authors assumed that the improved agro-chemical properties of the soil using biochar and vermicompost had a positive effect on plant growth, selected physiological parameters, and the tomato yield. In order to verify this hypothesis, the authors set up an experiment, which scrutinized the effect of biochar (CK0%; BA3, 3%; BA5, 5%; by mass of soil) and vermicompost (VA3, 3%; VA5, 5%) on photosynthesis, chlorophyll fluorescence, and tomato yield under greenhouse condition. A number of parameters specific to photosynthesis and chlorophyll fluorescence in tomato plants were analysed. The optimal parameter values were obtained in the treatment with the highest rate of vermicompost (VA5). The treatments with BA registered lower values, but these were higher, however, than those with CK. In summary, the authors highlight that for one season of tomato production, the application of 3% vermicompost is considered economical with regard to improving photosynthesis, enhancing the WUE, and increasing the tomato yield.

### 2.6. Phosphor and Potassium Use Efficiency

Besides N, phosphorus is the second most important nutrient in agriculture. The need to improve the use of P from fertilizers by crops stems from two basic factors: (i) the limited resources of raw materials economically viable for exploitation; (ii) the adverse effects of the component’s dispersion into the environment [38]. Opportunities to improve the use of P from fertilizers can be explored in various ways. It is important to create not only the optimal conditions for the mobility of H_2_PO_4_^−^ ions (e.g., the soil pH) but also the right choice of doses and type of fertilizer. As the research of Santos et al. indicates [39], in order to improve the efficiency of P use, it is crucial to select the right variety to suit the environment/location. The authors investigated the additive and non-additive effects of commercially relevant traits for the popcorn crop (grain yield—GY, popping expansion—PE, and expanded popcorn volume per hectare—PV) in different conditions of phosphorus (P) availability in two locations in Rio de Janeiro State, Brazil. Six S7 lines previously selected (three efficient and responsive; and three inefficient and non-responsive for P use) were used as testers in crosses with 15 progenies from the fifth cycle of intrapopulation recurrent selection of UENF-14. The 90-hybrid analysis allowed the authors to determine the combination with the highest impact of dominance genes on performance and responsiveness in the use of phosphorus for the GY, PE, and PV traits.

Chlorine is an essential micronutrient for plants. Its content in soils used for agriculture is usually at a much higher level than the nutritional needs of plants. One of the reasons for this condition is the widespread use of potassium in the form of chloride salt (KCl). Excessive Cl content in the soil can reduce the yield and quality of many crops and thus reduce the efficiency of K from fertilizers. The species sensitive to excess chlorine in the soil include coffee plants. High concentrations of Cl are related to an increase in plant water, which favors an undesirable fermentation of coffee fruits [40]. A way to bypass the problem would be to use K_2_SO_4_. However, this fertilizer increases the cost of fertilization. In the article, the authors proposed a partial replacement of KCl with K_2_SO_4_ [40]. To achieve this, the authors investigated the effect of blends of KCl and K_2_SO_4_ fertilizers at different proportions and their influence on the yield, nutritional state, and chemical composition and quality of the coffee beverage. The research clearly shows that the K content in the leaves was not influenced by the application of blends of K fertilizer while the Cl content increased linearly with the KCl applied. Fertilization with KCl reduces the cup quality and the activity of the polyphenol oxidase, probably due to the ion Cl. Taking into account the yield of coffee plants, the optimal ratio of KCl and K_2_SO_4_ was 1:3. However, the highest score in the cup quality test was observed with 100% K_2_SO_4_.

## 3. Conclusions

Improving the use of nutrients from fertilizers (the FUE) is one of the most important goals of modern agriculture in the context of the increasing demand for food and the growing pressure on the environment. This Special Issue presents a number of possibilities and strategies to improve the FUE. According to the presented publications, most of the research focuses on the possibility of improving the use of N by plants through balanced fertilization. Only in a state of equilibrium between the supplies of N and other nutrients to the plant during the growing season is it possible to effectively exploit the yield potential of a cultivated plant. The balanced fertilization of plants is, therefore, the key to sustainable agricultural production. Balanced fertilization should be supported by other activities aimed at improving the FUE, such as shaping the optimal conditions for nutrient uptake, including the effective use of P and K from fertilizers, foliar fertilization, or the application of innovative fertilizers with a controlled release rate of nutrients and/or nitrification inhibitors. At the same time, the development of new technologies and fertilization strategies should be accompanied by progress in plant breeding that better utilizes natural and anthropogenic sources of nutrients.
